# Functional Relevance of Protein Glycosylation to the Pro-Inflammatory Effects of Extracellular Matrix Metalloproteinase Inducer (EMMPRIN) on Monocytes/Macrophages

**DOI:** 10.1371/journal.pone.0117463

**Published:** 2015-02-06

**Authors:** Heng Ge, Wei Yuan, Jidong Liu, Qing He, Song Ding, Jun Pu, Ben He

**Affiliations:** 1 Department of Cardiology, Ren Ji Hospital, School of Medicine, Shanghai Jiao Tong University, Shanghai, China; 2 Department of Cardiac Surgery, Ren Ji Hospital, School of Medicine, Shanghai Jiao Tong University, Shanghai, China; University of California, San Diego, UNITED STATES

## Abstract

**Background and Objective:**

Extracellular matrix metalloproteinase inducer (EMMPRIN) is an important pro-inflammatory protein involved in the cellular functions of monocytes/macrophages. We have hypothesized that high-level heterogeneousness of protein glycosylation of EMMPRIN may have functional relevance to its biological effects and affect the inflammatory activity of monocytes/macrophages.

**Methods:**

The glycosylation patterns of EMMPRIN expressed by monocytes/macrophages (THP-1 cells) in response to different extracellular stimuli were observed, and the structures of different glycosylation forms were identified. After the purification of highly- and less-glycosylated proteins respectively, the impacts of different glycosylation forms on the pro-inflammatory effects of EMMPRIN were examined in various aspects, such as cell adhesion to endothelial cells, cell migrations, cytokine expression, and activation of inflammatory signalling pathway.

**Results:**

1) It was mainly the highly-glycosylated form of EMMPRIN (HG-EMMPRIN) that increased after being exposed to inflammatory signals (PMA and H_2_O_2_). 2) Glycosylation of EMMPRIN in monocytes/macrophages led to N-linked-glycans being added to the protein, with the HG form containing complex-type glycans and the less-glycosylated form (LG) the simple type. 3) Only the HG-EMMPRIN but not the LG-EMMPRIN exhibited pro-inflammatory effects and stimulated inflammatory activities of the monocytes/macrophages (i.e., activation of ERK1/2 and NF-κB pathway, enhanced monocyte-endothelium adhesion, cell migration and matrix metalloproteinase -9 expression).

**Conclusions:**

Post-transcriptional glycosylation represents an important mechanism that determines the biological effects of EMMPRIN in monocytes/macrophages. Glycosylation of EMMPRIN may serve as a potential target for regulating the inflammatory activities of monocytes/macrophages.

## Introduction

Inflammation is a key driving force in the pathophysiological process of many diseases，where activated monocytes/macrophages play a significant role [1–3]. Extracellular matrix metalloproteinase inducer (EMMPRIN)[4] is a transmembrane glycoprotein belonging to the immunoglobulin superfamily[5], originally identified as a surface protein of tumour cells that can stimulate the expression of matrix metalloproteinases (MMPs) in fibroblasts[6–8]. The implication of EMMPRIN in the activity of monocytes/macrophages has also been suggested. For instance, in a study on atherosclerosis, a well-recognized inflammatory disease[9], EMMPRIN expression found in human atheroma was mainly induced by monocytes/macrophages[4,10]. The functional roles of EMMPRIN in monocytes/macrophages seem to be well beyond stimulating MMPs, which may also include enhancing cell adhesion as well as migration into inflammation-involved tissues [11,12]—subsequent to the activation of inflammatory intercellular signally pathway, such as mitogen-activated protein kinases (MAPK) and NF-κB[12]. EMMPRIN is therefore considered an important protein involved in the inflammatory activity of monocytes/macrophages.

One of the most striking features of EMMPRIN is its heterogeneous glycosylation forms. The molecular weights of the protein can vary greatly, even to a difference of ~30 kDa between a highly-glycosylated (HG) and a less-glycosylated (LG) form[5]. Results from studies on tumour cells show that both HG- and LG-EMMPRIN are characterized by N-linked glycosylation [13–15]. However, the glycan structures can differ: the LG form comprises simple-type glycans of a high-mannose composition, while the HG form features a complex-type carbohydrate structure[13,14]. The heterogeneous glycosylation of EMMPRIN has also been observed in monocytes/macrophages[16]. *In vitro* experiments have revealed significant increases of HG-EMMPRIN during monocytes’ differentiation into macrophages[16].

Differentiated glycosylation of EMMPRIN exhibits functional relevance in tumor cells. For instance, caveolin, a scaffolding protein composing the caveolae membrane, was shown to mediate tumour-suppressing effects by being selectively associated with LG-EMMPRIN but not HG-EMMPRIN[13]. The functional difference between HG- and LG-EMMPRIN in monocytes/macrophages has also been implied. Sluijter and colleagues [17], by examining carotid atherosclerotic specimens, once reported that HG-EMMPRIN was highly associated with unstable lesion whereas LG-EMMPRIN was abundantly present with stable plaques, indicating that different glycosylation forms expressed by monocytes/macrophages are related to plaque characteristics.

Notwithstanding, to our best knowledge, there is still a lack of research specifically focusing on the “biological meaning” of EMMPRIN glycosylation in monocytes/macrophages. Since the pro-inflammatory effects of EMMPRIN have largely been proved, the knowledge of the post-transcriptional modification, glycosylation, of this protein leading to functional implications of inflammatory cells requires to be unravelled.

From this background, the present study aims to: i) identify the expression patterns and structural differences of glycosylation forms of EMMPRIN in monocytes/macrophages; ii) elucidate the functional impacts of the EMMPRIN glycosylation forms on the inflammatory activity of monocytes/macrophages.

## Methods

### Reagents

Fetal bovine serum (FBS) and Medium 199 (M-199) were purchased from Gibco BRL (Life Technologies, NY). Phorbol 12-myristate 13-acetate (PMA), H_2_O_2_, peptide-N-glycosidase F (PNGase F), swainsonine, calcein-AM and Coomassie blue were acquired from Sigma-Aldrich (St. Louis, MO); Endoglycosidase H (Endo H) from Hoffmann-La Roche (Basel Switzerland); TNF-α from eBioscience (San Diego, CA); PD98059 from Cell Signaling (Danvers, MA). For protein purification, the Immunopure rprotein A IgG plus orientation Kit and the silver staining kit were provided by Pierce (Rockford, IL); and the monoclonal antibody against EMMPRIN (mAb-EMMPRIN) was produced by Genscript Corporation (Nanjing, China). For Western blot, the protein extraction kit was acquired from Beyotime (Haimen, China); mouse monoclonal antibodies against EMMPRIN, pERK1/2, pIκBα, ERK1/2, IκBα and rabbit polyclonal antibodies against MMP9 from Abcam (Cambridge, MA); the mouse monoclonal antibody against β-actin from Santa Cruz (Dallas, Tex).

### Cell cultures

The human monocytic cell line, THP-1 and human umbilical vein cells (HUVECs) were acquired from Shanghai Institutes for Biological Sciences, Chinese Academy of Sciences. The THP-1 cells were cultured in the RPMI 1640 medium supplemented with 10% FBS, 10 mM HEPES, 10,000 U/ml penicillin and 10 mg/ml streptomycin, while HUVECs were grown in M-199 medium containing 10% FBS and antibiotics. Both were maintained at 37°C in a 5% CO_2_ incubator.

### Purification of EMMPRIN

EMMPRIN was purified from the membrane fraction of THP-1 cell by immunoaffinity chromatography using mAb-EMMPRIN as previously described[15]. The THP-1 cells were stimulated with PMA (100 ng/ml) for 24 hours, after which, the cell membranes were extracted using the membrane protein extraction kit according to the manufacturer’s instructions. The supernatants of the extracts were applied to an immunoaffinity column which was coupled with mAb-EMMPRIN, and further stabilized with homobifunctional NHS-ester cross-linker DSS (all done according to the manufacturer’s instructions).

After recirculating the sample through the column for 12 hours at 4°C, the column was washed with Binding/Wash Buffer for three times, and the EMMPRIN protein was then eluted from the column with a solution of 50 mM diethylamine and 30 mM octylglucoside (pH 11.5). The eluted protein was immediately neutralized with 0.5 M NaH_2_PO_4_ followed by dialyzed (0.01 M PBS, pH 7.4) and lyophilized.

The purified protein was defined as highly glycosylated (HG-EMMPRIN). LG-EMMPRIN was derived by treating HG-EMMPRIN (100 μg/ml) with PNGase F (0.1 U) for 24 hours. Both HG-EMMPRIN and LG-EMMPRIN were verified by silver stained SDS-PAGE (12%) and Western blot assay.

### Western blot

After different treatments, THP-1 cells were rinsed with PBS and lysed with lysis buffer (50 mM Tris-HCl pH 8.0, 1 mM EDTA, 1% Triton X-100, 0.5% sodium deoxycholate, 0.1% SDS, 150 mM NaCl, 2x protease inhibitor mix). Cell lysates were separated by SDS-PAGE and transferred to PVDF membrane. After being blocked for 1 hour with 5% non-fat milk in TBS-T (Tris-buffered saline containing 0.1% Tween 20), the PVDF membrane was incubated with the primary antibody, followed by the secondary antibody. The blots were detected using the ECL Plus Western blotting detection system (GE Healthcare).

### Cell adhesion assay

HUVECs were stimulated with 10 ng/ml TNF-α for 6 hours. After dedicated treatments, THP-1 cells were stained with 5 mol/l calcein-AM at 37°C for 30 minutes before being added onto the HUVEC monolayers at a density of 10^6^ cells/ml. After 30 minutes, non-adherent monocytes were washed off. The attached monocytes were quantified by averaging the numbers of cells counted from six randomly selected optical fields under fluorescent microscopy for each treatment.

### Cell migration assay

The differently treated THP-1 cells were cultured in non-FBS medium in the upper chamber of the transwell insert (8μm pore size, BD) with a density of 1×10^6^ per well. The RPMI 1640 medium supplemented with 10% FBS was added to the wells below, which constituted the lower chambers of the chemotaxis assay.

After an incubation at 37°C in 5% CO_2_ for 6 hours, the filters were removed and the cells were stained by Giemsa stain. Cells were quantified by averaging the numbers counted from 6 high-power fields (200× magnification)

### Gelatin zymography

MMP activities were determined by gelatin zymography. 10^6^ cells were seeded in 60mm culture dishes with 3 ml culture medium. After 24 hours, cell-free cultural media were collected for assay. Each sample (30 μl) was resolved by SDS-PAGE using 10% polyacrylamide gel containing 0.1% gelatin. The gels were washed twice in 2.5% (w/v) Triton X-100 for 30 minutes and then incubated for 20 hours with a reaction buffer containing 50 mM Tris, 0.2 mM NaCl, 5 mM anhydrous CaCl_2_ and 2.5% Triton X-100. Afterwards, the gels were stained with 0.5% (w/v) Coomassie blue (R-250), and destained with a buffer consisting of 20% methanol, 10% acetic acid and 70% distilled water for 30 minutes, in order to visualize the zymogen bands. Finally, the zymography gels were scanned and analysed using special software (National Institutes of Health, USA).

### Statistical analysis

Data were expressed as Mean±S.E.M. Statistical analysis was performed by using one-way ANOVA test, the difference between specific group and the control group was then analysed using Student Newman Keuls Test, a p value < 0.05 was considered to be statistically significant. SPSS software version 22.0 (SPSS, Inc., Chicago, IL) was employed.

## Results

### Characterization of glycosylation forms of EMMPRIN in monocytes/macrophages

**Fig 1:**
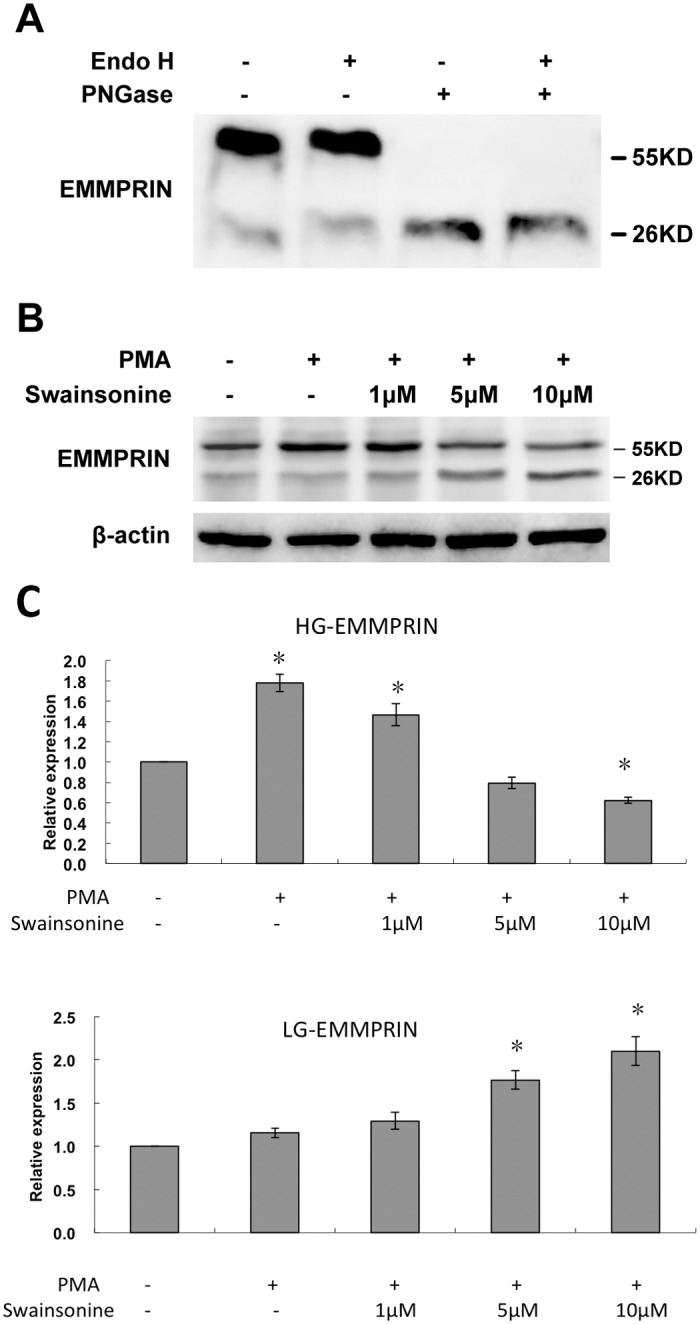
Characterization of glycosylation patterns of EMMPRIN in THP-1 cells. A: A representative Western blot of 3 independent experiments reveals two differently glycosylated EMMPRIN proteins from THP-1 cell, which refer to HG-EMMPRIN (with larger molecular weight) and LG-EMMPRIN (with smaller molecular weight). The protein glycosylation had different response to the treatment of PNGase F or Endo H. B: A representative Western blot demonstrates that the expression of HG-EMMPRIN by THP-1 cells responsive to PMA induction was inhibited by swainsonine. C: Quantitative analysis of 3 independent experiments probing the change of HG-EMMPRIN (upper figure) and LG-EMMPRIN (lower figure) in response to PMA and swainsonine. The band intensity was normalized to that of the β-actin. *P < 0.05, compared with the control group (whose value was set as 1.0).

The HG-EMMPRIN purified from the THP-1 cells was subjected to digestion by two types of endoglycosidases: PNGase F and Endo H[18], for that the former is capable of removing complex-type N-linked glycans from glycoproteins and the latter specifically target at simple-type N-linked glycans. After digestion by PNGase F, HG-EMMPRIN was no longer detectable; by contrast, Endo H mediated little effect on the presence of HG-EMMPRIN. (Fig. 1A). Such results can indicate that HG-EMMPRIN is mainly composed of complex-type, N-linked glycans.

To confirm our finding, we pre-treated THP-1 cells with swainsonine, an inhibitor of α-mannosidase being capable of preventing the formation of complex-type oligosaccharides for glycoprotein[19]. When HG-EMMPRIN expression was enhanced of by PMA induction, this expression significantly declined, in a concentration-dependent manner, with the treatment of swainsonine, (Fig. 1B and 1C). Interesting, an inverse increase in LG-EMMPRIN expression was concurrently observed.

### Changing patterns of EMMPRIN glycosylation in monocytes/macrophages

**Fig 2:**
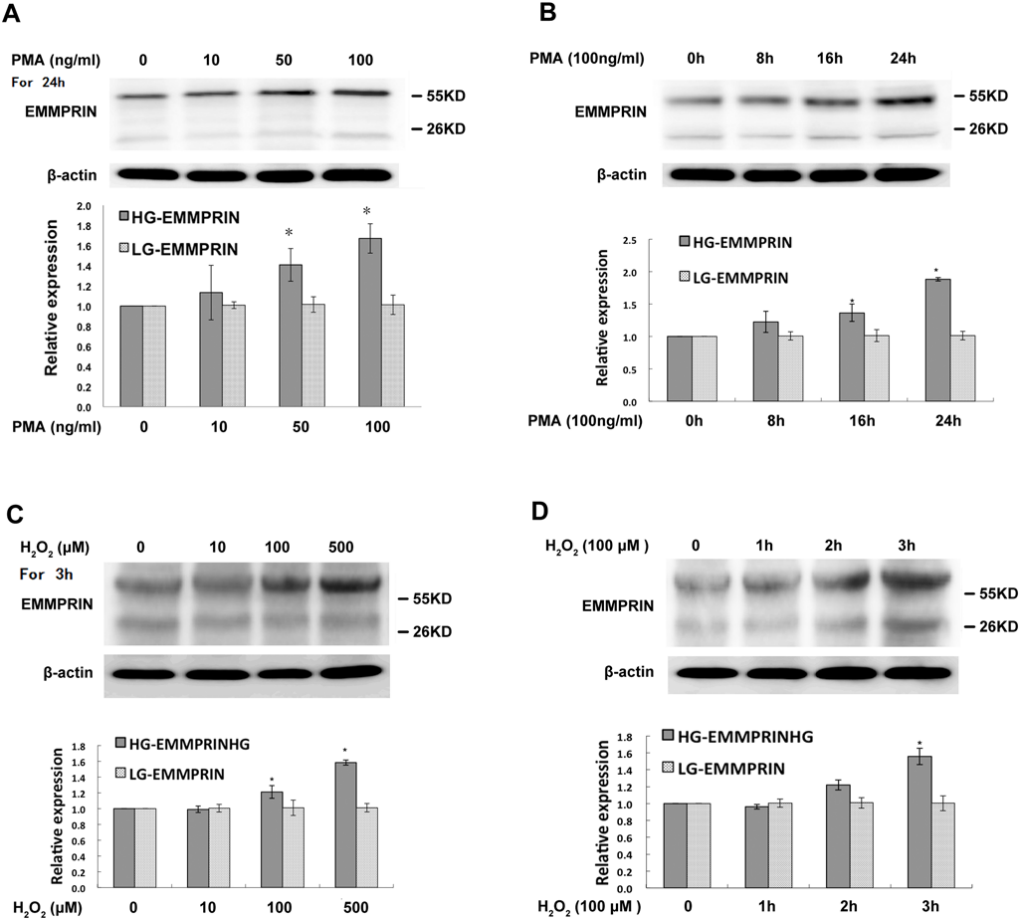
Changing of EMMPRIN glycosylation in response to pro-inflammatory stimuli in THP-1 cells. A representative Western blot demonstrates the changing of EMMPRIN glycosylation in response to PMA (A and B) or H_2_O_2_ (C and D) both in a concentration and time-dependent manner. Quantitative analysis of 3 independent experiments is shown for each treatment. The band intensity was normalized to that of theβ-actin. *P < 0.05, compared with the control group (whose value was set as 1.0).

To illustrate the changes in the glycosylation pattern of EMMPRIN in monocytes in response to extracellular stimuli, we monitored the expression levels of HG- and LG-EMMPRIN when the cells were exposed to PMA and H_2_O_2_, respectively. Previous reports have indicated that PMA can activate monocytes, resulting in their differentiation into macrophages, during which the expression of EMMPRIN can be greatly enhanced[16,20]. In our current study, it was mainly the expression of HG-EMMPRIN that increased, with little change identified in LG-EMMPRIN expression, and such an increase seemed to observe in both a concentration-dependent and a time-dependent way in response to PMA stimulation (Fig. 2, A and B). Similarly, after being treated with H_2_O_2_ (i.e. a stimulus of oxidative stress, another key pro-inflammatory factor activating monocytes[21]), the HG-EMMPRIN expression of the cells was also significantly increased, whereas the LG-EMMPRIN expression exhibited no difference (Fig. 2C and D). These results purport that HG-EMMPRIN is more responsive to inflammatory signals than the LG form in monocytes.

### Functional relevance of EMMPRIN glycosylation to inflammatory activities of monocytes/macrophages

In light of the fact that the glycosylation pattern of EMMPRIN in monocytes alters when activated by inflammatory signals, we furthered our study to investigate whether HG-EMMPRIN and LG-EMMPRIN can differently regulate the inflammatory activity of monocytes/macrophages. To do so, we examined several aspects of cellular functionality relating to inflammatory activities: i.e. adhesion to the vascular endothelium, cell migration and MMP-9 production.

**Fig 3:**
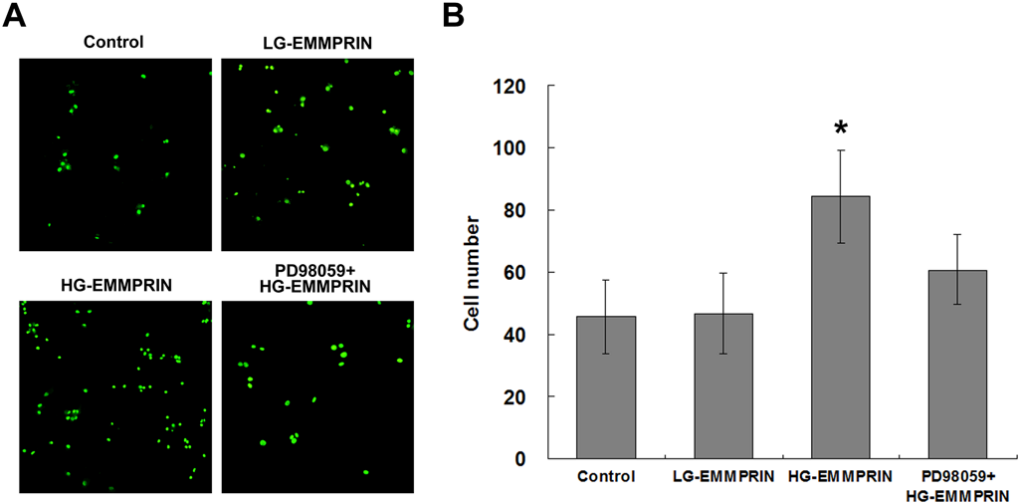
The impact of EMMPRIN glycosylation on monocytes-endothelium adhesion. A: Representative sections demonstrating the adhesive capabilities of fluorescently labelled THP-1 cells to the HUVEC monolayer when treated with HG-EMMPRIN (with or without pre-treatment of PD98059), LG-EMMPRIN or the control medium. B: Quantification of adhesive cell numbers of 3 independent experiments. *P < 0.05, compared with the control group.

In the monocytes-endothelium adhesion test, where the adhesion of THP-1 cells to HUVECs (as mediated by TNF-α)[22] was assessed, the treatment of HG-EMMPRIN (100μg/ml for 24 hours) led to a significantly higher number of cells attached than that resulting from the treatment of LG-EMMPRIN (100μg/ml for 24 hours) and the control (culture medium) (Fig. 3). LG-EMMPRIN treatment did not result in an increased number of attached cells when compared with the control.

**Fig 4:**
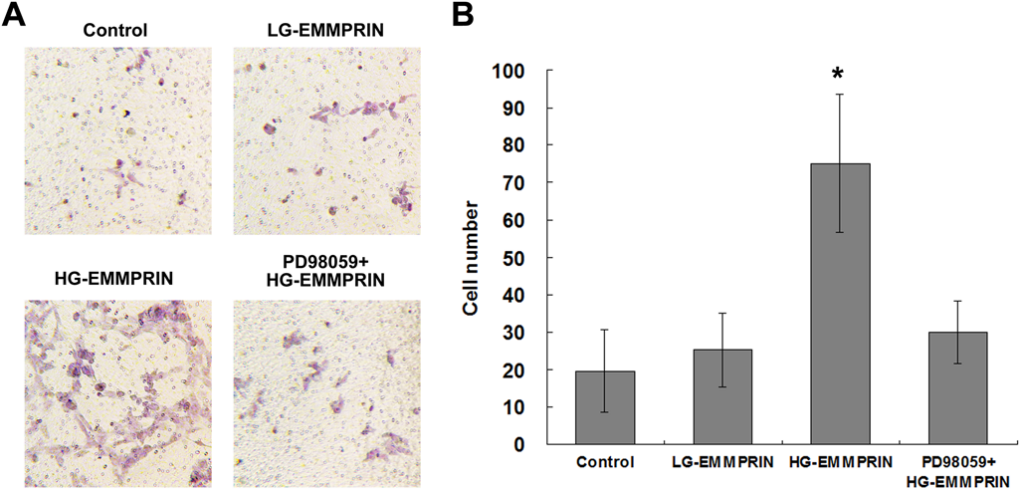
The impact of EMMPRIN glycosylation on monocytes migration. A: Representative sections demonstrating migratory activities of THP-1 cells in response to the treatment of HG-EMMPRIN (with or without pre-treatment of PD98059), LG-EMMPRIN or the control medium, respectively, as measured by the Boyden chamber assay. B: Quantification of migratory cell numbers of 3 independent experiments. *P < 0.05, compared with the control group.

In another experiment, the Boyden chamber assay[23] was applied to assess the migratory capability of THP1 cells corresponding to different glycosylation patterns of EMMPRIN. The cells were examined under the condition of LG-EMMPRIN treatment (100μg/ml), HG-EMMPRIN treatment (100μg/ml), or the control (culture medium). It was observed that the treatment of HG-EMMPRIN significantly enhanced the migration of THP-1 cells, while the LG-EMMPRIN treatment seemed to lack such an effect—with a level of migration comparable to that of the control (Fig. 4).

**Fig 5:**
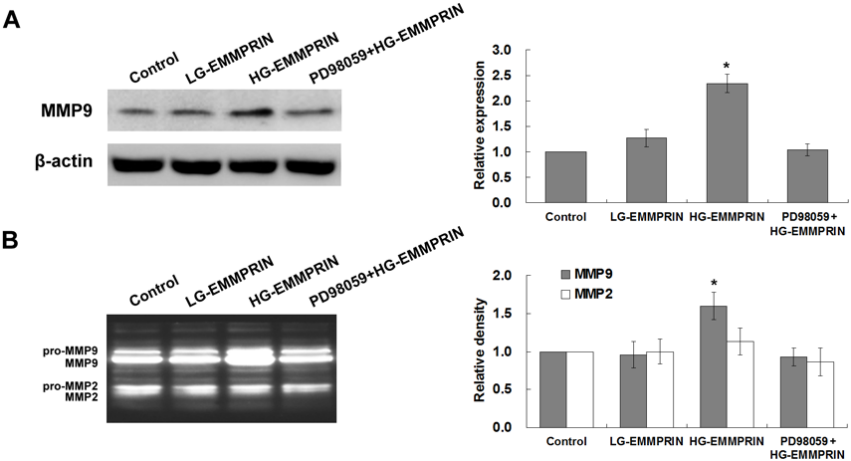
The impacts of EMMPRIN glycosylation on the expression and gelatinolytic activity of MMP-9 in THP1 cells. Representative Western blot (upper) and gelatine zymography (lower) results demonstrating the expression and the activity of MMP-9 by THP-1 cells in response to the treatment of HG-EMMPRIN (with or without pre-treatment of PD98059), LG-EMMPRIN or the control medium, respectively. Quantitative analysis of 3 independent experiments is shown on the right side. For Western blot, the band intensity was normalized to that of theβ-actin. *P < 0.05, compared with the control group (whose value was set as 1.0).

The next step was to identify whether glycosylation of EMMPRIN can also influence MMP expression in monocytes/macrophages. MMP-9 expression by monocytes is closely associated with the inflammatory effects of monocytes/macrophages (i.e. progression and instability of atherosclerotic plaque)[24] and can be induced by EMMPRIN[25]. Our Western blot analysis detected a remarkable increase of MMP-9 from the supernatants of the HG-EMMPRIN-treated cells (100μg/ml for 24 hours), compared with those treated with LG-EMMPRIN (100μg/ml for 24 hours) or the control (culture medium) (Fig. 5A). No difference between the MMP-9 levels was observed between the cells treated with LG-EMMPRIN and the control. In a similar way, gelatine zymography identified a higher level of MMP-9 activity from the cells treated with HG-EMMPRIN, whereas the activity exhibited by the cells treated with LG-EMMPRIN was comparable to that of the control (Fig. 5B). In the meanwhile, the expression of MMP-2, a type of MMP not responsive to EMMPRIN induction, was neither affected by HG-EMMPRIN treatment nor by LG-EMMPRIN treatment.

### HG-EMMPRIN activates the pro-inflammatory ERK/NF-κB signalling pathway

**Fig 6:**
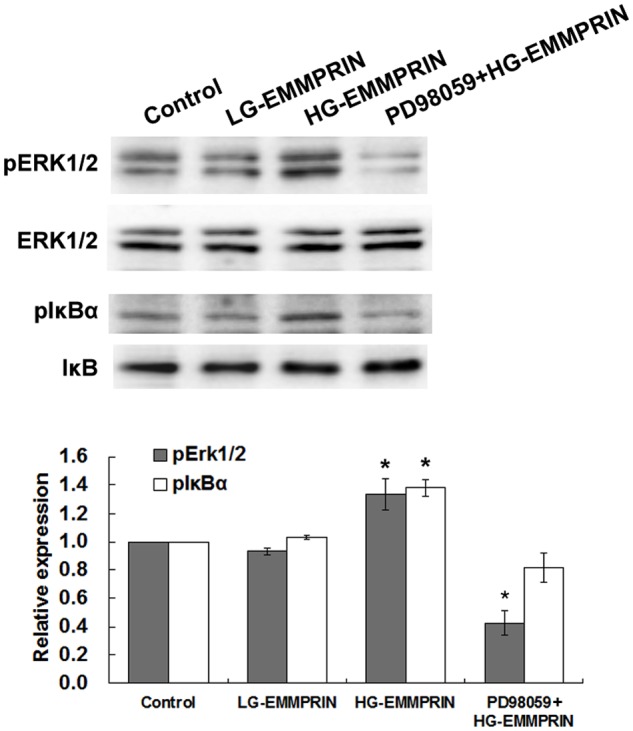
The impacts of EMMPRIN glycosylation on the activation of the ERK/NF-κB signalling pathway. A representative Western blot illustrates the intercellular phosphorylation of ERK1/2 and IκBα when THP-1 cells were treated with HG-EMMPRIN (with or without the pre-treatment of PD98059), LG-EMMPRIN or the control medium. Quantitative analysis of 3 independent experiments is shown below, the band density was normalized to that of the total ERK1/2 and IκBα, respectively. * *P* < 0.05 compared with the control group (whose value was set as 1.0).

It is conceivable that the inflammatory activities of monocytes/macrophages are driven by activation of intracellular pro-inflammatory signalling pathways. Our previous studies have highlighted the relationship between EMMPRIN and the activation of ERK/NF-κB pathway in monocytes/macrophages[12,26]. Therefore, we were interested to know whether the previous findings could be attributed to the glycosylation of EMMPRIN resulting in the activation of the signalling pathways. To do so, we examined the levels of phosphorylated ERK1/2 (i.e. pERK1/2) and phosphorylated IκBα (i.e. pIκBα) in THP-1 cells. While the former can indicate the levels of activated MAPK *per se*, the latter refers to the degradation of a key inhibitor of NF-κB and hence the activation of NF-κB[27]. Unsurprisingly, higher levels of both phosphorylated forms were found by Western blot in the cells treated with HG-EMMPRIN (100μg/ml for 24 hours), in comparison with the phosphorylation levels observed in the LG-EMMPRIN (100μg/ml for 24 hours) treated cells which were just comparable to those of the controls (culture medium) (Fig. 6).

Addition of an MEK inhibitor, PD98059[28], to the cells further verified the critical role of HG—EMMPRIN in activating the ERK/NF-κB pathway. As such, pre-treatment of PD98059 (50μM, 2 hours) inhibited the elevated levels of phosphorylation of ERK1/2 and IκBα, and, in the meanwhile, significantly attenuated the enhanced monocyte-endothelium adhesion, monocyte migration and MMP-9 expression that were responding to the treatment of HG-EMMPRIN (Figs. 3–6).

## Discussion

In this study, we have identified a posttranslational modification mechanism, i.e. glycosylation, of the transmembrane protein EMMPRIN that underlies its functional role in inflammation. We have demonstrated that the highly-glycosylated form of EMMPRIN rather than its less-glycosylated form is associated with heightened inflammatory responses by monocytes/macrophages. We have further illustrated that the HG-form is essential for EMMPRIN to drive the inflammatory activity of monocytes/macrophages by activating intracellular pro-inflammatory signalling and subsequently enhancing cell adhesion, cell migration and MMP-9 production. This is the first study to date that systemically differentiates the biological functions of heterogeneous glycosylation forms of EMMPRIN in monocytic cells.

Both laboratory and clinical studies have previously indicated the implication of EMMPRIN in diseases driven by the inflammatory activities of monocytes/macrophages. For instance, Schmidt and colleagues have linked EMMPRIN to the development of atherosclerosis by demonstrating an upregulation of EMMPRIN expression in circulating monocytes among patients with acute myocardial infarction (a severe clinical setting usually caused by inflammation-provoked plaque rupture[25]). A potential mechanism has been suggested, which points to the key roles this molecule play in recruiting monocytes/macrophages into atherosclerotic lesions and enhancing their inflammatory activities[10,11,16,25]. Our previous results also showed that EMMPRIN expression was enhanced during monocytes’ differentiation into macrophages[16,20], and the suppression of EMMPRIN expression was paralleled by significantly reduced inflammatory activity of monocytes/macrophages[12].

However, the preceding studies were mostly focused on EMMPRIN *per se*, without probing into the functional relevance of its glycosylation variation, in spite of its well-observed structural differences. The only clue to the implication of differentially glycosylated EMMPRIN in monocytes/macrophage-induced inflammatory disease was derived from Sluijter and colleagues’ work[17], which linked HG-EMMPRIN with atherosclerotic plaque instability. Their findings are in line with our current results, in that: i) inflammatory or oxidative stress, two specific micro-surroundings within advanced or unstable lesions, can significantly increase HG-EMMPRIN in monocytes/macrophages. ii) HG-EMMPRIN but not LG-EMMPRIN is associated with an enhanced monocytic inflammatory activity that may lead to plaque instability.

The importance of the glycosylation of EMMPRIN in exerting its pro-inflammatory effects in monocytes/macrophages has been systematically investigated by our study. The HG form seems to be essential for EMMPRIN to mediate key aspects of inflammatory activity of monocytes/macrophages[29–31]. In our study, the cells stimulated by HG-EMMPRIN were better able to adhere to the vascular endothelium and migrate towards pro-inflammatory stimuli; and they also exhibited enhanced expression of inflammatory cytokines such as MMP-9. On the contrary, the effects of LG-EMMPRIN were only comparable to the control. Being consistent with previous findings that implicated the roles of ERK and NF-κB in pro-inflammatory signalling of EMMPRIN [12,26,32], we have further proved that it is the HG form of EMMPRIN but not the LG form that can activate these signalling pathways, accounting for the induction of inflammatory activity of monocytes/macrophages by EMMPRIN.

Among all the findings of this study is the confirmation of the glycosylation structures of EMMPRIN in monocytes/macrophages. Similar to what were observed in tumour cells[13–15], the glycosylation forms are characterized by N-glycosylation, and the HG-form comprises complex-type carbohydrates and the LG-form contains simple-type glycans. Although the results were well expected, this knowledge can still yield important insights. It is not far-fetched to consider the complex type of N-glycosylated EMMPRIN as a potential target for inhibition of the over-activated inflammatory activity of monocytes/macrophages, from which new therapeutics employing cutting-edge glycoengineering technologies may arise [33,34].

## Conclusion

The highly-glycosylated form is essential for EMMPRIN protein to exert its pro-inflammatory effects in monocytes/macrophages. The glycosylation of EMMPRIN in monocytes/macrophages can be considered a potential target for development of new therapeutics that is able to inhibit over-activated inflammatory activity of monocytes/macrophages
